# Effects of Exogenous Tannase and Papain on the Flavor Quality of Black Tea During Fermentation

**DOI:** 10.3390/foods15101729

**Published:** 2026-05-14

**Authors:** Xin Lei, Chen Li, Li-Xian Wang, Su-Nan Huang, Xin-Feng Jiang

**Affiliations:** 1 Cash Crops Research Institute of Jiangxi Province, Nanchang 330006, China; leixintea@163.com (X.L.); hanwuji1110@126.com (C.L.); wanglixian0214@126.com (L.-X.W.); 2 College of Biological and Environmental Engineering, Jingdezhen University, Jingdezhen 333000, China; 18221939071@163.com; 3 Jiangxi Provincial Key Laboratory of Plantation and High Valued Utilization of Specialty Fruit Tree and Tea, Nanchang 330202, China

**Keywords:** black tea, exogenous enzymes, fermentation, metabolomics, volatile compounds, aroma molecular docking, tea flavor chemistry

## Abstract

This study systematically investigated the application effects of exogenous enzymes (tannase/papain) during black tea fermentation, aiming to optimize flavor. Tannase treatment significantly reduced astringent substances like tea polyphenols and enhanced 11 metabolites, including Myricetin-3-O-arabinoside, thereby effectively enhancing fruity and sweet aromas. Papain treatment increased free amino acids and accumulated Myricetin-3-O-arabinoside and Vitexin-7-O-glucoside, significantly enhancing umami and floral flavors. Both enzyme treatments increased the odor activity value (OAV) of key aroma compounds such as (Z)-3-Hexen-1-ol and 1-Hexanol. Molecular docking revealed that hydrophobic interactions and hydrogen bonding are the key driving forces for aroma compound binding, OR5M3 and OR1A1 are key olfactory receptors for black tea aroma perception, and Benzene, n-butyl- was identified as a key aroma compound for the studied receptors. Overall, exogenous enzyme technology significantly changed the flavor of traditional black tea by reducing bitterness and astringency, as well as enhancing sweetness and floral aroma.

## 1. Introduction

As the most widely consumed tea globally, black tea is highly favored for its characteristic “red liquor and red leaves” and its mellow, brisk, and rich aroma. Black tea is produced through processes such as withering, rolling, fermentation and drying, while the most critical process is fermentation [1]. The fermentation step is pivotal to the quality formation of black tea. It involves the oxidative polymerization of tea polyphenols, catalyzed mainly by endogenous polyphenol oxidase (PPO) and peroxidase (POD), which yields theaflavins, thearubigins, and characteristic aroma components [2]. In recent years, the application of exogenous enzymes to enhance fermentation efficiency and flavor quality has become a significant direction in black tea processing research. Currently, exogenous enzyme fermentation technology has demonstrated significant effects on black tea processing techniques and quality, potentially leading to time savings. Reports from existing studies have indicated that the appropriate application of *β*-glucosidase can facilitate the hydrolysis of tea liquor glycosides, leading to the release of elevated levels of free aromatic metabolites, such as benzyl alcohol and phenylethyl alcohol, thereby enhancing the fruity and floral aroma profile [3]. Solid-state fermentation of black tea utilizing the fungus *Eurotium cristatum* has been reported to reduce the bitterness and astringency of the tea liquor while conferring a characteristic fungal aroma [4]. The synergistic application of cellulase, pectinase, laccase, and *β*-glucosidase significantly enhanced the aroma of aged Wuyi Rock Tea, resulting in a 1.14-fold increase compared to the control [5]. The protein from Se-enriched tea treated with papain demonstrated enhanced free radical scavenging activity [6]. Therefore, profoundly exploring the mechanism of flavor modulation by exogenous enzymes in black tea holds significant importance for advancing innovation in black tea processing technology.

Tannase is recognized as an important biotechnology-relevant enzyme, utilized in numerous industrial applications, including the manufacture of instant tea, beer, fruit juices, certain wines, and the production of gallic acid [7]. Tannase catalyzes the hydrolysis of ester and gallate bonds within gallotannins [8], thereby releasing gallic acid and glucose, which in turn facilitates the conversion of bitter and astringent ester-type catechins into non-ester-type catechins [9]. This process mitigates bitterness and astringency without compromising the nutritional integrity of tea infusions and beverages. Additionally, it enhances clarity and, to a certain extent, promotes the dissolution of nutrients, thereby elevating overall product quality [10]. According to the findings of Liu et al. [8], during the formation of ripe Pu’er tea, tannase hydrolyzes catechin and theaflavin gallates, releasing gallic acid and catechins. Furthermore, the tannase hydrolysates were reported to enhance the antioxidant activity of the tea.

As a protease, papain, owing to its proteolytic activity and specificity, is widely applied in numerous industrial fields, including meat tenderization, dairy processing, beer brewing, pharmaceuticals, cosmetics, and textiles [11]. Papain possesses the capability to hydrolyze peptide bonds at the carboxyl terminus of L-lysine, L-arginine, L-citrulline, and glycine residues within proteins, leading to the release of a greater variety of small peptides or free amino acids. The protein content in tea accounts for approximately 22% of the dry matter weight, but only about 1% of it is water-soluble protein. Over 90% of the protein is insoluble and cannot directly contribute to the taste of the tea infusion. Through the action of proteases, the insoluble protein in tea can be hydrolyzed into various amino acids, which helps enhance the aroma and freshness of the tea infusion. Moreover, it can hydrolyze animal and plant tissues into bioactive peptides which exhibit functions such as immune promotion, lowering blood pressure, and antioxidant activities [12].

In the field of medicine, due to its anti-inflammatory, hemolytic, anticoagulant, debridement and antibacterial properties [13], papain has become a high-value biological product in multiple industries. It can be seen that the practical value of tannase in tea production has been confirmed. However, it is noteworthy that the application potential of papain, a highly efficient protease, has not yet been effectively explored in the field of tea processing. To fill this research gap, conducting a systematic and in-depth study on the changes in flavor caused by both substances during the fermentation of black tea is of great significance for the development of new tea processing techniques.

Molecular docking technology, by simulating at the molecular level, reveals the interaction between olfactory receptors and volatile metabolites. It is notable that this technology has been successfully applied to the identification mechanisms of key aroma components such as osmanthus green tea [14], Longjing [15], Pu’er tea [16], and Zunyi black tea [17], fully demonstrating that it is an effective means for exploring the formation mechanism of tea flavors.

This study conducted a fermentation process of black tea using tannase and papain enzymes, systematically analyzing its sensory quality, key metabolites (volatile/non-volatile) and the flavor formation mechanism. The research combined multivariate statistics and KEGG (Kyoto Encyclopedia of Genes and Genomes) analysis to reveal the influence of exogenous enzymes on metabolic pathways; subsequently, through OAV and molecular docking, the interaction mechanism between key aroma metabolites and olfactory receptors was clarified. This work provides a theoretical basis for using exogenous enzyme technology to improve the quality of black tea in a targeted manner.

## 2. Materials and Methods

### 2.1. Materials and Reagents

Methanol, glacial acetic acid, formic acid and acetonitrile (HPLC) were purchased from Thermo Fisher (Thermo Scientific, Waltham, MA, USA). Standards were purchased from Shanghai Yuanye Biotechnology Co. (Shanghai, China). Purified water (18.2 MX) was prepared in a Millipore Mill-Q Ultrapure Water System (Billerica, MA, USA). All other reagents and solvents were of analytical grade and purchased from China National Pharmaceutical Group Corporation (Beijing, China). Tannase-treated (DN) and papain-treated (MGDB) groups were purchased from XiaSheng (Beijing, China) Biotechnology Development Co., Ltd.

### 2.2. Tea Processing and Exogenous Enzyme Treatments

This study was conducted during the spring of 2023. Fresh tea leaves (one bud and two leaves) were harvested from 22-year-old Fudingdabai tea plants, a national cultivar (No. GS13001—1985). The samples were collected from the ecological tourism tea garden base of Jiangxi Economic Crops Research Institute in Nanchang City, China (116.01° E, 28.37° N).

**Figure 1:**
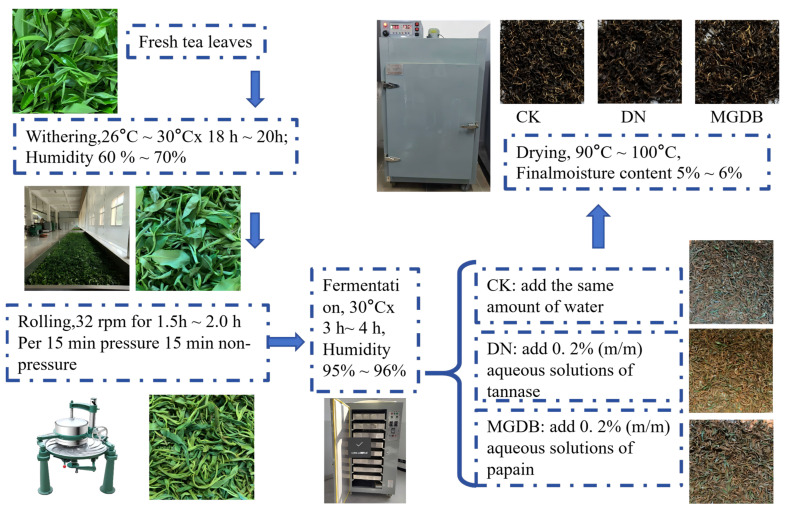
The schematic diagram of black tea processing. Note: CK, without additives. DN, with tannase added. MGDB, with papain added. All other treatments remain the same.

The fresh leaves were divided into three equal portions, and each treatment was prepared in triplicate. Black tea was processed following the standard stages of withering, rolling, fermentation, and drying. During the fermentation process, the control group (CK) received no exogenous enzymes. The tannase-treated group (DN) and papain-treated group (MGDB) were supplemented at the beginning of fermentation with 0.2% (*w*/*w*) aqueous solutions of tannase and papain, respectively, with the total addition volume not exceeding 10% of the fresh sample weight. All enzyme preparations were food-grade additives sourced from Sunson Industry Group Co., Ltd. (Beijing, China). Between drying steps, the samples were fully cooled to room temperature and rehydrated; specific parameters are shown in Figure 1. The dried tea samples were stored in a −20 °C freezer for subsequent analysis.

### 2.3. Sensory Evaluation

According to the Chinese National Standard GB/T 23776-2018 [18], sensory quality assessment was conducted on three groups of tea samples. Five sensory testers, each with over 10 years of evaluation experience and professional training, evaluated the color, taste and aroma of the tea infusion in a professional sensory evaluation room.

### 2.4. Color Measurement

With the tea infusion prepared the same as that in the sensory evaluation, the Konica Minolta (Tokyo, Japan) CR-400 colorimeter was used to measure infusion colors according to the CIELAB parameters: L* represents brightness, with higher values indicating a lighter color; a* represents greenness (−) and redness (+); b* represents blueness (−) and yellowness (+); C represents purity or saturation, with higher values indicating a purer color; and H represents hue, with the value ranging from 0° to 360°, where 0°, 90°, 180°, and 270° represents red, green, blue and magenta, respectively.

### 2.5. Determination of Characteristic Tea Chemical Composition

The water extract of tea samples was determined according to Chinese National Standard GB/T 8305-2013, and the contents of free amino acids, tea polyphenols, caffeine and tea pigments were measured by spectrophotometry [19]. In addition, caffeine and eight monomeric catechins were quantitatively analyzed using High-Performance Liquid Chromatography (HPLC) following specific methods detailed in the references [20].

### 2.6. Analysis of Non-Volatile Metabolites in Black Teas Using UPLCMS/MS

#### 2.6.1. Extraction of Non-Volatiles

The tea samples were vacuum freeze-dried and then ground into powder. A mass of 50 mg of each sample was weighed, and of 70% aqueous methanol extraction solvent containing the internal standard, pre-cooled to −20 °C, was added. This was followed by vortex mixing for 30 s, repeated six times with a 30 min interval between each mixing. The mixture was then centrifuged (at 12,000 rpm for 3 min), and the supernatant was aspirated. The sample was filtered using a microporous membrane (with a pore size of 0.22 μm) and the filtered sample was stored in the sample vial for UPLC-MS/MS analysis.

#### 2.6.2. LC-MS Conditions

LC conditions: chromatographic column: agilent SB-C18 1.8 µm, 2.1 mm * 100 mm; mobile phase: Phase A is ultra-pure water (with 0.1% formic acid added) and Phase B is acetonitrile (with 0.1% formic acid added); elution gradient: 5% B proportion at 0.00 min, linearly increases to 95% within 9.00 min, and remains at 95% for 1 min, then drops to 5% from 10.00 to 11.10 min, and reaches 5% again for 14 min; flow rate 0.35 mL/min; column temperature 40 °C; injection volume 4 μL.

MS conditions: electrospray ionization (ESI) temperature 550 °C; ion spray voltage (IS) 5500 V (positive ion mode)/−4500 V (negative ion mode); ion source gas I (GSI), gas II (GSII), and curtain gas (CUR) are set to 50, 60, and 25 psi respectively, and the collision-induced ionization parameters are set to high. QQQ scanning uses MRM mode, and the collision gas (nitrogen) is set to medium. Through further optimization of declustering potential (DP) and collision energy (CE), the DP and CE of each MRM ion pair were determined. Based on the metabolites eluted in each period, a specific set of MRM ion pairs was monitored in each period.

#### 2.6.3. Metabolite Qualitative and Quantitative Analysis of LC-MS

Based on the self-built database MWDB (MetWare database), metabolites were qualitatively identified using secondary spectral information. During the analysis, isotope signals were removed, and duplicate signals of K^+^, Na^+^ and NH_4_^+^ ions, as well as duplicate signals of fragment ions of substances that are themselves other larger molecular weight substances, were also eliminated. Specific methods followed the procedure referenced in this paper [21].

### 2.7. Analysis of Volatile Metabolites in Black Teas Using GC-MS

#### 2.7.1. Extraction of Volatiles

The tea samples were ground into powder using liquid nitrogen, and 500 mg of each was placed in a 20 mL headspace bottle. The bottle contained a saturated sodium chloride solution and 20 μL of 3-hexanone-2,2,4,4-d4 (as an internal standard solution) at a concentration of 10 μg/mL.

#### 2.7.2. HS-SPME Extraction Conditions

Each sample vial was agitated for 5 min at 60 °C. A 120 µm DVB/CWR/PDMS extraction head was inserted into the headspace bottle of the sample. The headspace extraction was conducted for 15 min. The sample was then analyzed at 250 °C for 5 min, followed by GC-MS separation and identification. The extraction head was aged at 250 °C in the Fiber Conditioning Station for 5 min before sampling.

#### 2.7.3. GC-MS Conditions

GC conditions: DB-5MS capillary column (30 m × 0.25 mm × 0.25 μm, Agilent J&W Scientific, Folsom, CA, USA), carrier gas is high-purity helium (purity not less than 99.999%), constant flow rate 1.2 mL/min, injection port temperature 250 °C, non-split injection, and solvent delay 3.5 min. Temperature program: 40 °C for 3.5 min, then increase to 100 °C at 10 °C/min, then to 180 °C at 7 °C/min, finally to 280 °C at 25 °C/min, and maintain for 5 min.

MS conditions: electron impact ion source (EI), ion source temperature 230 °C, quadrupole temperature 150 °C, mass spectrometry interface temperature 280 °C, electron energy 70 eV, scanning mode is selected as ion detection mode (SIM), and qualitative and quantitative ion precise scanning (GB 23200. 8-2016 [22]).

#### 2.7.4. Metabolite Identification and Quantification Principles of GC-MS

Based on multiple species, the literature, partial standards, and retention indices, an independent database was established. The specific qualitative method referred to in Reference [23] was used. Quantitative ions were selected for the integration and correction of chromatographic peaks to enhance the accuracy of quantification.

### 2.8. OAV Calculation

The OAV of each volatile metabolite are calculated by dividing the concentration by the odor threshold. The odor threshold data are derived from previous studies [24], while the odor descriptions are referenced from the database (https://www.thegoodscentscompany.com/, http://perflavory.com/, http://www.odour.org.uk/odour/index.html and http://foodflavorlab.cn/#/home).

### 2.9. Molecular Docking

Using the molecular docking analysis method proposed by Sun and Z [17], and employing the Autodock Vina 1.1.2 software, the binding interactions between the selected key aroma metabolites and the olfactory receptors were studied. The docking results were further analyzed and visualized using Discovery Studio v.2019 to assess potential binding modes and key molecular interactions. The olfactory receptors studied included OR1A1 (UniProt ID: Q9P1Q5), OR1D2 (UniProt ID: P34982), OR1G1 (UniProt ID: P47890), OR2W1 (UniProt ID: Q9Y3N9), and OR5M3 (UniProt ID: Q8NGP4).

### 2.10. Statistical Analysis

All chemical tests were repeated three times, and the LC-MS and HS-SPME-GC-MS analyses of each sample were conducted in three parallel determinations. The results were expressed as the mean ± standard deviation. Significance analysis was performed using SPSS 27, with a *p*-value < 0.05 set as the level of statistical significance. The heatmap was generated using TBtools-II software version 2.376. Multivariate statistical analysis of metabolomics data (UPLC-MS/MS and GC-MS) was processed through the MetWare Cloud platform (https://cloud.metware.cn/).

## 3. Results

### 3.1. Sensory Characteristics of Black Tea Fermented with Tannase and Papain

**Figure 2:**
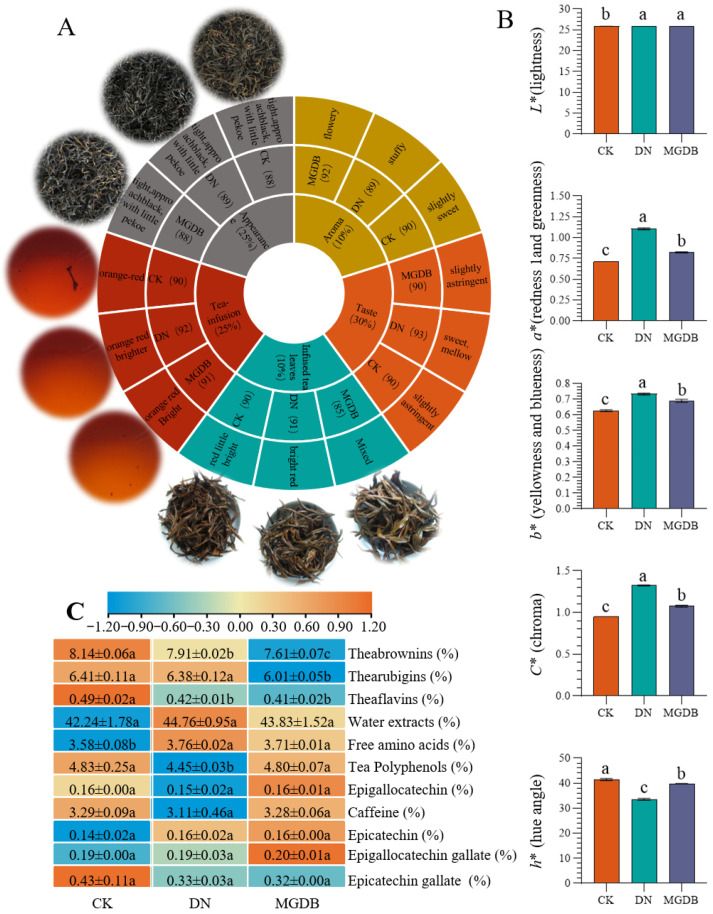
(**A**) Sensory characteristics in CK, DN and MGDB. (Infusion color, infused leaves, appearance, taste and aroma.) (**B**) Color parameters. (**C**) Content of characteristic components in CK, DN and MGDB (different letters indicate significant differences, *p* < 0.05). Each row represents a metabolite, and each column represents a sample. Color intensity indicates the relative expression level of each metabolite, with orange representing high expression and blue representing low expression.

The sensory evaluation results (Figure 2A) showed that the traditional black tea control sample (CK) had an orange–red liquor color, a slightly sweet aroma, a slightly astringent taste, a tight appearance with visible trichomes, and a dark, lustrous color. The DN group was significantly superior to CK in terms of liquor color, sweet and mellow taste, and spent leaves, but received a lower score for aroma, presenting a stuffy flavor. (MGDB achieved a higher aroma score, characterized by a floral aroma, but its spent leaves score was reduced to 85 points, and its taste was consistent with CK. Measurement of the tea infusion color difference (Figure 2B) showed that the L*, a*, b*, and C* values of both DN and MGDB samples were significantly higher than those of the traditional black tea samples, representing that the addition of tannase and papain would make the tea soup brighter, redder, and yellow, with purer colors, and the hue value H* of the traditional tea was more green. After the addition of tannase, the overall taste of the tea infusion is significantly improved. This could be attributed to the fact that tannase can more thoroughly hydrolyze polyphenolic substances, effectively reducing their astringent taste, while promoting a more complete fermentation process, thus making the tea color and leaf base appear more red and bright [25]. While papain mainly hydrolyzes the proteins in the tea leaves to release more amino acids; these amino acids are not only the source of the freshness and liveliness of the tea infusion, but can also serve as aroma precursors, further transforming into rich volatile aroma substances, thus helping to enhance the overall aroma quality.

### 3.2. Characteristic Metabolites in Black Tea Fermented with Tannase and Papain

Tea quality is highly correlated with its chemical composition [26]. Therefore, 16 specific components unique to tea were determined, including water extracts, tea polyphenols, free amino acids, caffeine, three tea pigments, and eight catechins (Figure 2C). Compared with CK, it was found that the free amino acids in DN and MGDB significantly increased (*p* < 0.05), providing a fresh taste to the tea infusion and being positively correlated with the umami of the tea infusion [27]. Papain is a proteolytic enzyme that can accelerate the accumulation of amino acids during fermentation. Furthermore, the contents of water extracts, four catechins, and caffeine showed no significant change (*p* < 0.05; Figure 2C). Future studies involving taste threshold determination and sensory evaluation of specific amino acids will further clarify their targeted impact on the sensory refinement of black tea.

Water extracts can serve as an indicator of the richness of substances in tea infusions, while caffeine is one of the indicators for bitterness [27,28], the results showed no significant difference (*p* < 0.05). It can be seen that the addition of these two enzymes has little or no effect on the water extracts and caffeine. Tea polyphenols are abundant metabolites in tea plants. They are responsible for conferring the characteristic astringency and bitterness to the tea infusion and serve as the major functional components of tea [26]. The content of the four detected catechins did not show a significant difference among the three samples, suggesting that the fermentation process during production may have been sufficiently thorough, leading to the complete conversion of catechins and, consequently, no obvious variance. However, the content of tea polyphenols in DN was significantly lower than that in CK and MGDB. Combined with the contents of the four catechins, this indicates that the DN sample had a lower catechin content, which resulted in a reduction in bitterness. Tannase can catalyze the hydrolysis of ester bonds and glycosidic bonds in gallic catechins, releasing gallic acid and glucose [25]. This further proves the evaluation results. The three tea pigment components—TFs, TRs, and theabrownins (TBs)—are the oxidation products of catechins and are the main pigment components responsible for the tea liquor color [29].

The contents of TFs and TRs in the DN sample were significantly reduced (*p* < 0.05), and the contents of all three components (TFs, TRs, and TBs) in the MGDB sample were also significantly reduced (*p* < 0.05). During black tea fermentation, galloylated catechins are first oxidized by PPO to produce O-quinone intermediates, which are subsequently converted into TFs. This is followed by the synergistic action of PPO and POD, which catalyzes the conversion of quinones, TFs, and their derivatives into TRs [30,31]. The formation of TBs primarily relies on the synergistic effect of extracellular enzymes and the moist–hot environment during the pile-fermentation stage of black tea production [25]. It is speculated that the addition of these two enzymes may accelerate the conversion of catechins to tea pigments to some extent, and it may also combine with other metabolites to form more complex substances, further affecting the accumulation of tea pigments.

### 3.3. Non-Volatile Identification and Analysis

**Figure 3:**
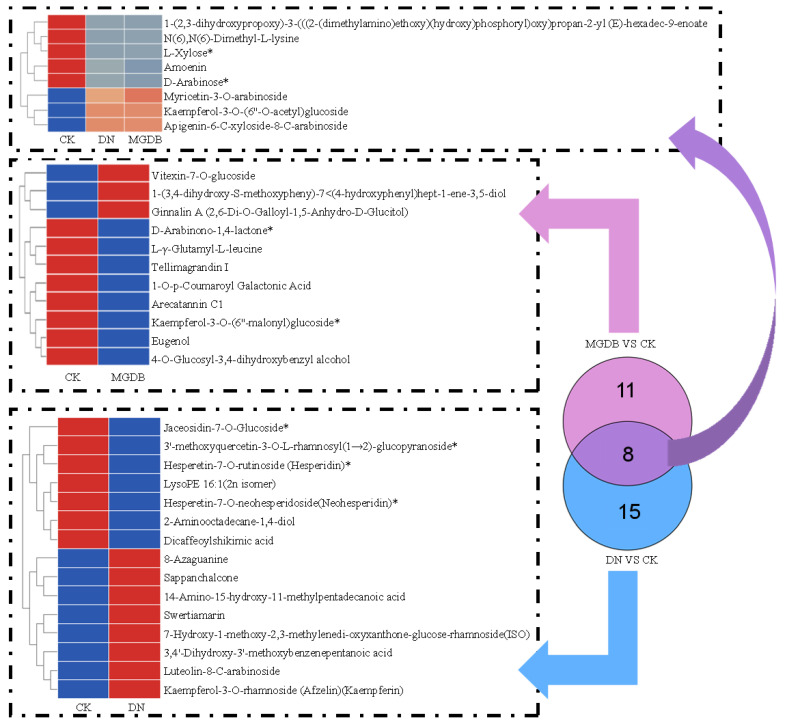
Heatmaps and Venn diagrams of key non-volatile substances in CK, DN, and MGDB. Each row represents a metabolite, and each column represents a sample. Color intensity indicates the relative expression level of each metabolite, with red representing high expression and blue representing low expression.

To further investigate the effects of tannase and papain on black tea, LC-MS analysis was conducted on 891 common metabolites. Principal Component Analysis (PCA) showed that there was a clear separation between the repeated samples of CK, DN, and MGDB during the fermentation process (Figure S1A,B), indicating that the addition of exogenous enzymes during the fermentation process led to significant changes in the composition of metabolites. Comparing the metabolites of CK and DN, a total of 23 key metabolites were screened (Figure 3) using selection criteria of VIP (Variable Importance in Projection) ≥ 1 and FC (Fold Change) ≥ 2 or FC ≤ 0.5. Among these, the relative abundances (RLs) of 11 metabolites (VIP > 1, *p* < 0.05 and FC > 2) significantly increased, including Sappanchalcone, Luteolin-8-C-arabinoside, and Myricetin-3-O-arabinoside, etc. These metabolites mainly fall into six categories: flavonoids (six types), nucleotides and their derivatives (one type), lipids (one type), terpenoids (one type), phenolic acids (one type), and others (one type); among them, 12 components decreased (VIP > 1, *p* < 0.05, FC < 0.5), such as Jaceosidin-7-O-Glucoside*, 3′-methoxyquercetin-3-O-L-rhamnosyl(1→2)-glucopyranoside*, Hesperetin-7-O-rutinoside (Hesperidin)*, etc. These components include flavonoids (five metabolites), lipids (three metabolites), carbohydrates (two metabolites), phenolic acids (one metabolite), and amino acids and their derivatives (one metabolite). It is worth noting that after adding tannase, the content of Myricetin-3-O-arabinoside increased by 10.43-fold compared to the original level. Current research indicates that this metabolite holds high research value for its activity in scavenging DPPH and inhibiting α-glucosidase [32]. To further analyze the biological significance of these differential metabolites, functional annotation and pathway analysis were conducted. This study was analyzed using the KEGG database. The enrichment analysis mapped 28 metabolites to 14 metabolic pathways, primarily involving the biosynthesis of secondary metabolites, the biosynthesis of flavonoids, and other related metabolic processes (Figure S1C). No pathways showed significant enrichment (*p* < 0.05).

Comparing (CK) with MGDB, a total of 19 key differential metabolites were identified. Thirteen key metabolites decreased (VIP > 1, *p* < 0.05, FC < 0.5), such as D-Arabinono-1,4-lactone*, Eugenol, Kaempferol-3-O-(6″-malonyl)glucoside*, etc. These components include phenolic acids (three metabolites), carbohydrates (three metabolites), tannins (two metabolites), flavonoids (two metabolites), amino acids and their derivatives (two metabolites), and lipids (one metabolite). Two flavonoids showing the largest reduction were Amoenin (FC = 0.07) and Kaempferol-3-O-(6″-malonyl)glucoside (FC = 0.04). Six metabolites showed a significant increase (VIP > 1, *p* < 0.05 and FC > 2), such as Ginnalin A (2,6-Di-O-Galloyl-1,5-Anhydro-D-Glucitol), Myricetin-3-O-arabinoside, 1-(3,4-dihydroxy-S-methoxypheny)-7<(4-hydroxyphenyl)hept-1-ene-3,5-diol, Vitexin-7-O-glucoside, Kaempferol-3-O-(6″-O-acetyl)glucoside and Apigenin-6-C-xyloside-8-C-arabinoside. These include flavonoids (four metabolites), phenolic acids (one metabolite), and tannins (one metabolite). The concentrations of Vitexin-7-O-glucoside and Myricetin-3-O-arabinoside were approximately 12 times higher than in CK. Vitexin-7-O-glucoside is an antioxidant flavonoid with biological activities such as the treatment of hyperlipidemia, fatty liver, and protection of cardiac function [33]. Myricetin-3-O-arabinoside has certain additional antioxidant and anti-sugar effects [32]. Thus, the addition of papain can enhance the health benefits of black tea. To further analyze the biological significance of these differentially expressed metabolites, functional annotation and pathway analysis were performed using the KEGG database. Enrichment analysis mapped 19 metabolites to 9 metabolic pathways, and 2 pathways were significantly enriched (*p* < 0.05). These pathways mainly involve ascorbate and aldarate metabolism and Apigenin C-glycosides biosynthesis (Figure S1D).

### 3.4. Volatile Metabolites

#### 3.4.1. Identification and Analysis of Volatile Metabolites in DN and MGDB

**Figure 4:**
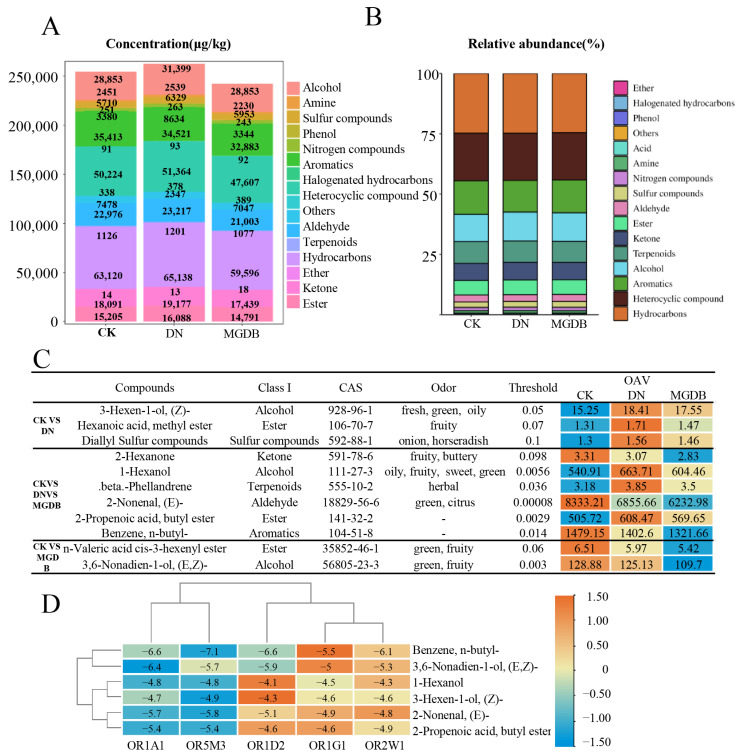
Categories, concentrations and OAV of volatile metabolites in CK, DN and MGDB. (**A**) Concentrations of volatile metabolites. (**B**) Relative abundances of volatile metabolites. (**C**) Odor activity values (OAV) of volatile metabolites and their aroma types. (“CK vs. DN” and “CK vs. MGDB” indicate the key differential aroma components (OAV > 1 and VIP > 1) identified in the respective pairwise comparisons. “CK vs. DN vs. MGDB” represents the common key differential aroma components among the three groups. The “OAV” column on the far right denotes the specific odor activity values for each component. (**D**) Heatmap of binding energies between key volatile compounds and olfactory receptors. The olfactory receptors include OR1A1, OR5M3, OR1D2, OR1G1, and OR2W1.

To further investigate the effects of tannase and papain application on the aroma of black tea, we identified a total of 417 volatile metabolites. These volatiles were mainly classified into 16 categories: acids (seven metabolites), aldehydes (29 metabolites), halogenated hydrocarbons (three metabolites), nitrogen metabolites (six metabolites), phenols (four metabolites), aromatics (25 metabolites), amines (seven metabolites), alcohols (40 metabolites), sulfur metabolites (nine metabolites), ethers (one type), others (one type), terpenoids (69 metabolites), hydrocarbons (54 metabolites), ketones (37 metabolites), heterocyclic metabolites (67 metabolites), and esters (58 metabolites). The total volatile metabolite contents of CK, DN and MGDB were 254,721.01 μg/kg, 262,701.92 μg/kg and 242,566.52 μg/kg, respectively (Figure 4A).

The DN samples showed a higher content of volatile metabolites (Figure 4B), thus resulting in rich and diverse aroma characteristics. The DN group mainly contained hydrocarbons (24.80%), heterocyclic metabolites (19.55%), aromatics (13.14%), and alcohols (11.95%), while the CK group was dominated by hydrocarbons (24.78%), heterocyclic metabolites (19.72%), and aromatics (13.90%). The dominant components in the MGDB group were hydrocarbons (24.57%), heterocyclic metabolites (19.63%), and aromatics (13.56%).

In conclusion, the analysis indicates that hydrocarbons, heterocyclic metabolites, aromatics, alcohols, and terpenoids all contribute to the black tea aroma, which is consistent with the results of previous black tea aroma studies [34]. Notably, compared to the CK group, the contents of alcohols, terpenoids, hydrocarbons, and heterocyclic metabolites in the tannase fermentation samples are higher, which may lead to a change in the proportion of the aroma, thereby changing the type of the aroma. This can be demonstrated by the sensory evaluation aroma of stuffy flavor. The addition of papain resulted in a decrease in the content of most aroma components in the sample, yet the resulting sensory aroma profile transformed into a floral type, which has certain research value for the targeted regulation of black tea flavor.

#### 3.4.2. The Role of Volatile Metabolites in Fermented Black Tea with Tannase and Papain

To further investigate the differences in volatile metabolites between black tea fermented with tannase and papain, two supervised Orthogonal Partial Least Squares Discriminant Analysis (OPLS-DA) models were constructed using multivariate statistical analysis to evaluate changes in volatile metabolite content (Figure S1E,F). The OPLS-DA analysis results indicated that the application of tannase and papain significantly altered the composition of volatile metabolites in black tea. Based on the VIP results from the OPLS-DA model, a total of 28 differential volatile metabolites (VIP > 1, *p* < 0.05; Table S1) were identified between the CK (control) and DN (tannase-treated) groups, and 38 differential volatile metabolites (VIP > 1, *p* < 0.05; Table S2) were identified between the CK and MGDB (papain-treated) groups. Of these, 12 metabolites were common to both comparisons.

According to the VIP results, the top five key volatile metabolites in CK and DN groups were: 1-Octanamine, N-methyl-, *β*-phellandrene, Dodecane, 3-methyl-, Benzenemethanol, alpha-methyl- alpha-(1-methyl-2-propenyl)-, 1,7-nonadiene, and 4,8-dimethyl- (Table S1). The top five key volatile metabolites in CK and MGDB were: Benzenemethanol, 3-hydroxy-, 2-octanone, 1,5-heptadien-4-ol, 3,3,6-trimethyl-, Naphthalene, decahydro-4a-methyl-1-methylene-7-(1-methylethenyl)-, [4aR-(4a. alpha., 7. alpha., 8a. beta. )]-, 7-oxabicyclo [4. 1. 0]heptan-2-one, and 6-methyl-3-(1-methylethyl)- (Table S2). Although key aroma substances have been identified, in the actual perception process, there is a minimum threshold for human perception of aroma molecules. When the concentration is below this threshold, the corresponding aroma components cannot be perceived. Therefore, through in-depth OAV analysis of volatile metabolites, the impact of tannase and papain on the characteristic volatile components of tea was evaluated.

#### 3.4.3. OAV Analysis of Differential Volatile Metabolites in DN and MGDB

The overall aroma characteristics of tea samples are determined by the content of volatile metabolites, the ratio of each component, and their odor thresholds. The odor threshold refers to the lowest concentration of volatile metabolites that can be perceived by the sense of smell. The OAV serves as an important indicator for evaluating the contribution intensity of volatile metabolites to the aroma. Currently, it is believed that volatile metabolites with an OAV greater than one have a significant contribution to the tea aroma [35]. Based on OAV analysis, this study utilized screening criteria of VIP greater than one, a *p*-value less than 0.05, and OAV greater than one, ultimately identifying 11 volatile metabolites with an OAV greater than one (Figure 4C), which were classified into seven categories: alcohols (three metabolites), esters (three metabolites), sulfur metabolites (one metabolite), ketones (one metabolite), terpenoids (one metabolite), aldehydes (one metabolite), and aromatics (one metabolite). Among these, six metabolites had an average OAV exceeding 10, namely: (E)-2-Nonenal (OAV = 7140.62), Benzene, n-butyl- (OAV = 1401.14), 1-Hexanol (OAV = 603.03), 2-Propenoic acid, butyl ester (OAV = 561.28), (E,Z)-3,6-Nonadien-1-ol (OAV = 121.24), and (Z)-3-Hexen-1-ol (OAV = 17.07).

Among the 11 volatile metabolites with OAVs greater than one, the OAVs of six key volatile metabolites were significantly increased in DN and MGDB, and the content in DN was higher than that in MGDB. The main characteristics were fresh, green, grassy, oily, fruity, horseradish-like, alcoholic, sweet, herbal, etc. The OAVs of five key volatile metabolites decreased in both DN and MGDB, mainly exhibiting flavors such as fruity, fungal, buttery, green, cucumber, and citrus. Among these, 2-Nonenal, (E)- itself possesses a grassy note, has an extremely low threshold, and its average OAV reached as high as 7140.62. However, its content significantly decreased after the addition of external enzymes, suggesting it may be a key aroma substance responsible for the grassy note in black tea. The addition of exogenous enzymes led to changes in the OAV of key flavor substances, and the changes in the CK of tannase and papain were consistent. When both increased simultaneously, the flavor substances in the tannase sample increased more; when both decreased simultaneously, the flavor substances in the papain sample decreased more. Thus, it can be seen that papain has a greater effect on the change in aroma.

### 3.5. Interaction of Key Aroma Metabolites with Olfactory Receptors

#### 3.5.1. Binding Energy of Key Aroma Metabolites with Olfactory Receptors

Olfactory receptors are a type of membrane protein that recognize odorant molecules and activate intracellular signaling pathways, thereby contributing to odor perception [36]. By performing molecular docking between olfactory receptors and aroma molecules, researchers not only verified the contribution of key flavor metabolites but also further revealed the flavor formation mechanisms of small molecule metabolites [15]. Five olfactory receptors with an established research foundation (OR1A1, OR1D2, OR1A2, OR2W1, OR5M3) [17] were selected for pairwise molecular docking with six key aroma metabolites. In each case, 19 cavities were selected for blind docking, and the result with the lowest binding energy was chosen as the final docking conformation The binding energies between the five olfactory receptors and the six volatile metabolites exhibited significant differences (Figure 4D), with binding energies ranging from −7.1 to −4.1 kcal/mol. with values ranging from −7.1 to −4.1 kcal/mol. Generally, negative binding energy values indicate spontaneous binding processes and stable ligand–receptor complex formation under simulated conditions [37,38,39].

Among the tested compounds, Benzene, n-butyl- (average OAV = 1401.14) exhibited the strongest binding affinity across all receptors, with binding energies ranging from −5.5 to −7.1 kcal/mol, particularly showing the lowest value with OR5M3 (−7.1 kcal/mol). In contrast, 1-Hexanol (average OAV = 603.03) and 3-Hexen-1-ol, (Z)- (average OAV = 17.07) displayed relatively weak binding affinities, with most values distributed between −4.1 and −4.9 kcal/mol, indicating less stable ligand–receptor interactions. 3,6-Nonadien-1-ol (E,Z), 2-Nonenal (E), and 2-Propenoic acid, butyl ester exhibited moderate binding affinities (approximately −5.0 to −6.0 kcal/mol).

This result suggests that aromatic hydrocarbons and unsaturated long-chain compounds (Benzene, n-butyl-; 3,6-Nonadien-1-ol (E,Z); 2-Nonenal (E)) tend to exhibit stronger binding affinities, whereas short-chain alcohols (1-Hexanol and 3-Hexen-1-ol (Z)) generally show weaker interactions with olfactory receptors, which is consistent with previous studies indicating that odorant–receptor binding is largely driven by hydrophobic interactions and depends on the physicochemical properties of ligands, such as molecular size, polarity, and structural features [40,41,42].

At the receptor level, OR5M3 and OR1A1 showed relatively stronger and more consistent binding affinities across multiple ligands, whereas OR1G1 and OR2W1 generally exhibited weaker interactions. For example, OR5M3 achieved the lowest binding energy for four out of the six compounds (−5.4 to −7.1 kcal/mol), indicating a potentially higher compatibility with structurally diverse odorants. This trend may be related to differences in the size, hydrophobicity, and flexibility of the receptor binding pockets, which have been reported to play a key role in determining ligand affinity and selectivity [40,43].

These results indicate that differences in the molecular structures of the key aroma metabolites lead to variations in the receptor binding regions, binding sites, and conformations. This difference affects their recognition ability and binding affinity. Therefore, receptors may selectively bind to volatile metabolites with strong binding affinity, thereby limiting the number of binding sites on the protein, but the binding of aroma is time-limited. Although the receptor will preferentially bind to volatile metabolites with strong binding affinity, it will still be bound by low binding affinity volatile metabolites with high concentration and long duration after the binding separation, thereby maintaining its aroma type. Although 2-Nonenal, (E)- is not the aroma substance with the highest binding energy with OR5M3, its OAV of 7140.62 has an impact that cannot be ignored on the flavor, whereas Benzene, n-butyl- showed the strongest binding but a comparatively lower OAV (1401.14). This observation is consistent with previous findings that odor perception is influenced not only by receptor binding affinity but also by odorant concentration, volatility, and sensory threshold [44,45,46,47].

Cluster analysis showed that OR1A1 and OR5M3 clustered into one group, while OR1D2, OR1G1, and OR2W1 clustered into another group. The key aroma metabolites Benzene, n-butyl-, and 3,6-Nonadien-1-ol, (E,Z)- clustered together, and the remaining four 1-Hexanol and 3-Hexen-1-ol, (Z)- clustered together. 2-Nonenal, (E)- and 2-Propenoic acid, butyl ester clustered together. Through clustering, it was discovered that the aroma descriptions of 1-Hexanol (oily, fruity, sweet, green) and 3-Hexen-1-ol, (Z)- (fresh, green, oily) both contained similar characteristics such as “oily” and “green”. It is speculated that in the case of sufficient data, metabolites with the same aroma type can cluster together. The clustering method based on binding energy can be further used for the classification of aroma types of olfactory receptors, such as distinguishing between floral aroma receptor categories, etc.

#### 3.5.2. Types of Interaction Forces Between Floral Metabolites and Olfactory Receptors

**Figure 5:**
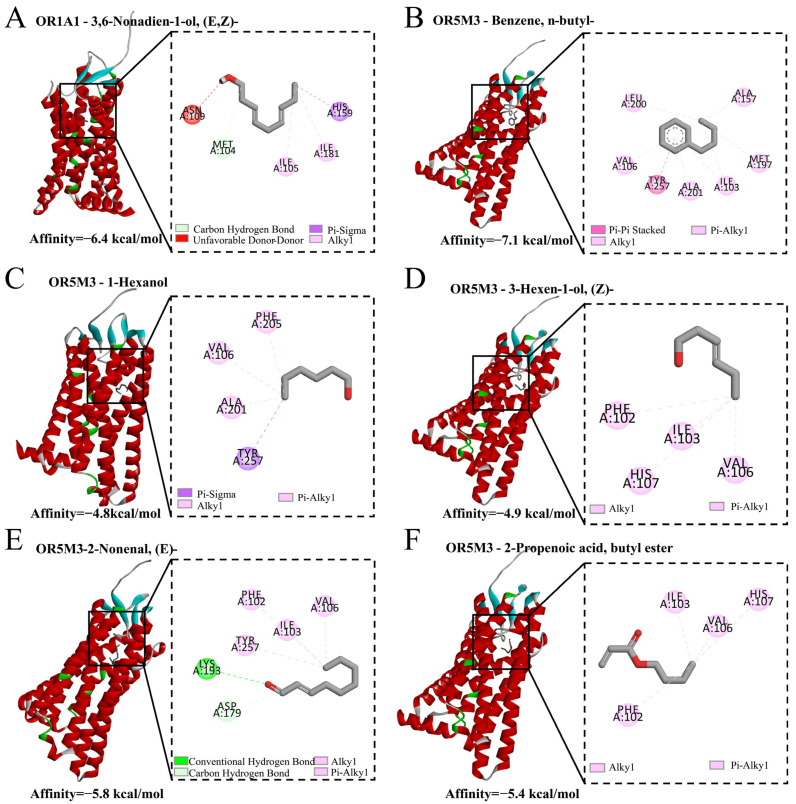
Molecular docking of floral aroma compounds to olfactory receptors. (**A**) OR1A1-3,6-Nonadien-1-ol, (E,Z)-. (**B**) OR5M3-Benzene, n-butyl-. (**C**) OR5M3-1-Hexanol. (**D**) OR5M3-3-Hexen-1-ol, (Z)-. (**E**) OR5M3-2-Nonenal, (E)-. (**F**) OR5M3-2-Propenoic acid, butyl ester.

Table S3 and Figure 5 show the results of molecular docking simulations, displaying the top five lowest-energy receptor complexes for each of the six aroma compounds: 1-Hexanol with OR5M3, 2-Nonenal, (E)- with OR5M3, 2-Propenoic acid, butyl ester with OR5M3, Benzene, n-butyl-with OR5M3, 3,6-Nonadien-1-ol, (E,Z)- with OR1A1, and 3-Hexen-1-ol, (Z)- with OR5M3. In this study, hydrophobic interactions were consistently found in all six groups of complexes, involving amino acid residues such as TYR, ALA, VAL, PHE, ILE, and LEU (Table S3, Figure 5). This is in agreement with previous studies indicating that hydrophobic interactions play a central role in odorant recognition, as most volatile aroma compounds are relatively nonpolar and tend to interact with hydrophobic binding pockets of olfactory receptors [40,41,42,45].

Hydrogen bonds were also an important driver of interactions between volatile compounds and olfactory receptors, suggesting their potential contribution to binding specificity and stability. Notably, in the OR1A1 complex with 3,6-Nonadien-1-ol, (E,Z)-, a hydrogen bond was formed with MET with bond length of 3.54 Å. Similarly, in the OR5M3 complex with 2-Nonenal, (E)-, hydrogen bonds were formed with LYS and ASP, with bond lengths of 3.29 Å and 3.48 Å, respectively. Hydrogen bonding has been widely reported to enhance ligand recognition specificity and stabilize odorant–receptor complexes, particularly for compounds containing polar functional groups such as carbonyl moieties (e.g., 2-Nonenal (E); 3,6-Nonadien-1-ol (E,Z)) [40,45,48].

To sum up, the differences in binding energies of aroma compounds to various olfactory receptors primarily stem from the diversity of these receptors, the chemical structures of the compounds [40,41,42].

These factors collectively influence the binding of aroma compounds to specific olfactory receptors, resulting in differences in binding affinity and interaction patterns. Compounds exhibiting relatively strong binding affinities and stable interaction patterns with olfactory receptors may play a more prominent role in odor recognition. When considered together with the flavor analysis results, these docking findings provide supportive evidence for the potential involvement of the identified compounds in the overall aroma characteristics of red tea.

## 4. Conclusions

In this study, we applied two exogenous enzymes during the fermentation stage of black tea processing, which led to a series of notable alterations in the final product quality. In tannase-treated black tea (DN), the contents of theabrownins (TBs), theaflavins (TFs), and tea polyphenols were significantly reduced, thereby alleviating the astringent taste. Metabolomic analysis identified 23 key metabolites, of which 11 showed elevated levels. The content of Myricetin-3-O-arabinoside reached 10 times that of traditional black tea, while the OAV of key aroma substances such as 3-Hexen-1-ol (Z-) and 1-Hexanol and 2-Propenoic acid, butyl ester also increased, thereby enhancing the fruity and sweet aromas of the tannase-treated black tea to a certain extent.

In papain-treated black tea (MGDB), the contents of the three primary tea pigments were reduced, while the level of free amino acids increased, contributing to a fresher and more refreshing taste. Metabolomic analysis identified 29 key metabolites, with 6 metabolites showing increased levels. Specifically, Myricetin-3-O-arabinoside and Vitexin-7-O-glucoside reached 12 times the concentrations found in traditional black tea. Additionally, the OAVs of aroma substances such as 1-hexanol, *β*-phellandrene, and butyl ester 2-propenoic acid increased, optimizing the fruity and sweet aroma attributes of MGDB.

Moreover, molecular docking results revealed that hydrophobic interactions were observed in all six ligand groups, while hydrogen bonds served as a crucial driving force for the interactions between volatile metabolites and olfactory receptors. We identified OR5M3 and OR1A1 as potential key olfactory receptors in black tea aroma perception and determined that Benzene, n-butyl- was a key aroma metabolite for the receptors studied.

Overall, we utilized exogenous enzyme technology to inoculate tannase and papain during the fermentation process of black tea, effectively enhancing the sweetness and floral aroma characteristics of the tea infusion, and identifying the relevant characteristic metabolites. Our results provide a theoretical foundation for further regulating the fermentation process with exogenous enzymes, especially by using tannase and papain to improve the quality of black tea. In the future, it is promising to explore the effects of other exogenous enzymes on the fermentation metabolism of black tea, thereby revealing the internal mechanism by which exogenous enzymes enhance the quality of black tea.

## Data Availability

The original contributions presented in this study are included in the article/Supplementary Materials; further inquiries can be directed to the corresponding author.

## Supplementary Materials

The following supporting information can be downloaded at: https://www.mdpi.com/article/10.3390/foods15101729/s1, Table S1: Concentrations of key volatile compounds in CK and DN; Table S2: Concentrations of key volatile compounds in CK and MGDB; Table S3: Binding energies between key volatile compounds and olfactory receptors. Figure S1: (A). OPLS-DA score plot of non-volatile substances in CK and D. (B). OPLS-DA score plot of non-volatile substances in CK and MGDB. (C). KEGG enrichment plot of differential non-volatile metabolites between CK and DN. (D). KEGG enrichment plot of differential non-volatile metabolites between CK and DN. (E). OPLS-DA score plot of volatile substances in CK and DN. (F). OPLS-DA score plot of volatile substances in CK and DN.
