# Supplementary material for: Effects of Exogenous Tannase and Papain on the Flavor Quality of Black Tea During Fermentation
Source: Foods. 2026 May 14;15(10):1729. doi: 10.3390/foods15101729 (PMC13205800; doi:10.3390/foods15101729)
Supplement: Supplementary file 1 [file foods-15-01729-s001.zip › Table S1 S2 S3.pdf]

**Table S1.** Concentrations of key volatile compounds in CK and DN

| Compounds | Class I | CAS | CK | DN | VIP | Fold_Change |
|---|---|---|---|---|---|---|
| Benzenemethanol, .alpha.-methyl-.alpha.-(1-methyl-2-propenyl)- | Alcohol | 61967-11-1 | 0.01±0.00 | 0.01±0.00* | 2.04 | 1.63 |
| Dodecane, 3-methyl- | Hydrocarbons | 17312-57-1 | 0.01±0.00 | 0.02±0.00** | 2.04 | 1.55 |
| Tricyclo[3.3.1.1(3,7)]decane, 2-nitro- | Nitrogen compounds | 54564-31-7 | 0.04±0.01 | 0.05±0.00* | 1.94 | 1.41 |
| (E)-2,6-Dimethylocta-2,5,7-trien-4-one | Ketone | 33746-72-4 | 0.02±0.00 | 0.03±0.00** | 1.97 | 1.30 |
| Hexanoic acid, methyl ester | Ester | 106-70-7 | 0.09±0.02 | 0.12±0.02 | 1.83 | 1.28 |
| 2-Oxepanone | Ester | 502-44-3 | 0.09±0.01 | 0.12±0.01* | 1.95 | 1.26 |
| Dicyandiamide | Nitrogen compounds | 461-58-5 | 0.16±0.01 | 0.21±0.02* | 1.91 | 1.25 |
| Allyl Sulfur compounds | Sulfur compounds | 27817-67-0 | 1.50±0.08 | 1.86±0.16* | 1.96 | 1.21 |
| 1-Hexanol | Alcohol | 111-27-3 | 3.03±0.17 | 3.72±0.28* | 1.96 | 1.20 |
| 5,6,7,8-Tetrahydroquinoxaline | Heterocyclic compound | 34413-35-9 | 0.55±0.07 | 0.67±0.10 | 1.75 | 1.19 |
| .beta.-Phellandrene | Terpenoids | 555-10-2 | 0.11±0.01 | 0.14±0.01 | 2.06 | 1.18 |
| 3-Hexen-1-ol, (Z)- | Alcohol | 928-96-1 | 0.76±0.06 | 0.92±0.10 | 2.00 | 1.18 |
| 2-Propenoic acid, butyl ester | Ester | 141-32-2 | 1.47±0.08 | 1.76±0.09* | 1.92 | 1.18 |
| Diallyl Sulfur compounds | Sulfur compounds | 592-88-1 | 0.13±0.00 | 0.16±0.01* | 1.87 | 1.17 |
| Pyrrole | Heterocyclic compound | 109-97-7 | 0.22±0.01 | 0.26±0.03 | 1.73 | 1.15 |
| Cyclohexanol | Alcohol | 108-93-0 | 0.20±0.02 | 0.22±0.02 | 1.77 | 1.11 |
| 1-OctanAmine,N-methyl- | Amine | 2439-54-5 | 0.10±0.01 | 0.11±0.01 | 2.08 | 1.10 |
| Isobornyl formate | Ester | 1200-67-5 | 1.50±0.16 | 1.45±0.14 | 2.00 | 0.95 |
| Benzene, n-butyl- | Aromatics | 104-51-8 | 20.71±1.45 | 19.64±1.23 | 1.64 | 0.93 |
| Benzene, (methylthio)- | Sulfur compounds | 100-68-5 | 1.00±0.11 | 0.94±0.10 | 2.00 | 0.92 |
| 2-Hexanone | Ketone | 591-78-6 | 0.32±0.03 | 0.30±0.03 | 1.80 | 0.91 |
| 3-Hexanone | Ketone | 589-38-8 | 0.01±0.00 | 0.01±0.00 | 1.89 | 0.89 |
| Octane, 2,3,3-trimethyl- | Hydrocarbons | 62016-30-2 | 0.31±0.02 | 0.28±0.02 | 1.73 | 0.89 |
| 1,5-Heptadien-4-one, 3,3,6-trimethyl- | Terpenoids | 546-49-6 | 0.17±0.02 | 0.16±0.01 | 2.00 | 0.89 |
| 2-Nonenal, (E)- | Aldehyde | 18829-56-6 | 0.67±0.03 | 0.55±0.08 | 1.87 | 0.80 |
| 1,7-Nonadiene, 4,8-dimethyl- | Hydrocarbons | 62108-28-5 | 0.04±0.00 | 0.03±0.00* | 2.04 | 0.77 |
| 5-Hepten-2-ol, 6-methyl- | Alcohol | 1569-60-4 | 0.04±0.00 | 0.03±0.01 | 1.80 | 0.77 |
| Undecane, 4,4-dimethyl- | Hydrocarbons | 17312-68-4 | 0.07±0.01 | 0.05±0.01 | 1.79 | 0.69 |

Note: The asterisk (*) in the same row indicates significant differences.(* $p < 0.05$ ** $p < 0.01$)

**Table S2.** Concentrations of key volatile compounds in CK and MGDB

| Compounds | Class I | CAS | CK | MGDB | VIP | Fold_Change |
|---|---|---|---|---|---|---|
| Bicyclo[3.2.1]oct-2-ene, 3-methyl-4-methylene- | Terpenoids | 49826-53-1 | 0.05±0.01 | 0.07±0.00* | 1.64 | 1.50 |
| Dodecane, 3-methyl- | Hydrocarbons | 17312-57-1 | 0.01±0.00 | 0.01±0.00* | 1.71 | 1.41 |
| 2-Octanone | Ketone | 111-13-7 | 0.01±0.00 | 0.01±0.00* | 1.88 | 1.20 |
| Allyl Sulfur compounds | Sulfur compounds | 27817-67-0 | 1.50±0.08 | 1.71±0.09* | 1.76 | 1.16 |
| Dicyandiamide | Nitrogen compounds | 461-58-5 | 0.16±0.01 | 0.18±0.01 | 1.64 | 1.16 |
| 2-Propenoic acid, butyl ester | Ester | 141-32-2 | 1.47±0.08 | 1.65±0.07* | 1.79 | 1.15 |
| 1-Hexanol | Alcohol | 111-27-3 | 3.03±0.17 | 3.38±0.16 | 1.73 | 1.14 |
| .beta.-Phellandrene | Terpenoids | 555-10-2 | 0.11±0.01 | 0.13±0.00 | 1.85 | 1.12 |
| 1-Propanone, 1-cyclopropyl- | Ketone | 6704-19-4 | 0.09±0.01 | 0.10±0.00 | 1.64 | 1.11 |
| Benzene, (methylthio)- | Sulfur compounds | 100-68-5 | 1.00±0.11 | 0.91±0.04 | 1.75 | 0.93 |
| 4-Hexen-1-ol, 5-methyl-2-(1-methylethenyl)-, (R)- | Terpenoids | 498-16-8 | 0.61±0.07 | 0.55±0.01 | 1.72 | 0.92 |
| 1,5-Heptadien-4-ol, 3,3,6-trimethyl- | Terpenoids | 27644-04-8 | 0.92±0.09 | 0.82±0.01 | 1.88 | 0.91 |
| 4-Penten-1-ol, propanoate | Ester | 30563-30-5 | 0.32±0.04 | 0.28±0.01 | 1.62 | 0.91 |
| Benzene, n-butyl- | Aromatics | 104-51-8 | 20.71±1.45 | 18.50±0.22 | 1.66 | 0.91 |
| 2-Hexanone | Ketone | 591-78-6 | 0.32±0.03 | 0.28±0.01 | 1.71 | 0.88 |
| 3,6-Nonadien-1-ol, (E,Z)- | Alcohol | 56805-23-3 | 0.39±0.03 | 0.33±0.01* | 1.72 | 0.87 |
| Butanethioic acid, 3-methyl-, S-(1-methylpropyl) ester | Ester | 2432-91-9 | 0.37±0.05 | 0.31±0.01 | 1.74 | 0.86 |
| 2-Propenoic acid, pentyl ester | Ester | 2998-23-4 | 0.03±0.00 | 0.02±0.00** | 1.66 | 0.86 |
| n-Valeric acid cis-3-hexenyl ester | Ester | 35852-46-1 | 0.39±0.05 | 0.33±0.02 | 1.67 | 0.86 |
| Ascaridole | Terpenoids | 512-85-6 | 0.03±0.00 | 0.02±0.00* | 1.76 | 0.81 |
| 7-Oxabicyclo[4.1.0]heptan-2-one, 6-methyl-3-(1-methylethyl)- | Ketone | 5286-38-4 | 0.08±0.01 | 0.06±0.00* | 1.86 | 0.81 |
| 2,6-Octadienal, 3,7-dimethyl-, (Z)- | Terpenoids | 106-26-3 | 0.15±0.02 | 0.12±0.01 | 1.78 | 0.81 |
| Imidodicarbonic diamide | Nitrogen compounds | 108-19-0 | 0.17±0.02 | 0.13±0.01 | 1.78 | 0.80 |
| (E)-2,6-Dimethylocta-2,5,7-trien-4-one | Ketone | 33746-72-4 | 0.02±0.00 | 0.02±0.00** | 1.73 | 0.79 |
| 5-Hexenal, 4-methylene- | Hydrocarbons | 17844-21-2 | 0.05±0.00 | 0.04±0.00** | 1.75 | 0.77 |
| Naphthalene, decahydro-4a-methyl-1-methylene-7-(1-methylethenyl)-, [4aR-(4a.alpha.,7.alpha.,8a.beta.)]- | Terpenoids | 17066-67-0 | 0.14±0.01 | 0.11±0.00* | 1.86 | 0.77 |
| 2-Nonenal, (E)- | Aldehyde | 18829-56-6 | 0.67±0.03 | 0.50±0.04** | 1.71 | 0.76 |
| Undecane, 2-methyl- | Hydrocarbons | 7045-71-8 | 0.59±0.06 | 0.44±0.03* | 1.80 | 0.76 |
| Cyclohexanol, 1-methyl-4-(1-methylethyl)-, trans- | Terpenoids | 3901-93-7 | 0.65±0.07 | 0.48±0.03* | 1.74 | 0.76 |

| | | | | | | |
|---|---|---|---|---|---|---|
| .beta.-Guaiene | Terpenoids | 88-84-6 | 0.07±0.00 | 0.05±0.01* | 1.65 | 0.75 |
| Benzenemethanol, 3-hydroxy- | Alcohol | 620-24-6 | 0.02±0.00 | 0.01±0.00* | 1.89 | 0.74 |
| 6-Nonen-1-ol, (E)- | Alcohol | 31502-19-9 | 0.31±0.03 | 0.23±0.01* | 1.73 | 0.74 |
| p-Mentha-1,5-dien-8-ol | Terpenoids | 1686-20-0 | 1.10±0.08 | 0.79±0.02** | 1.82 | 0.73 |
| (5R,10R)-10-Methyl-6-methylene-2-(propan-2-ylidene)spiro[4.5]dec-7-ene | Terpenoids | 28908-27-2 | 0.12±0.01 | 0.09±0.01** | 1.79 | 0.72 |
| 1,5-Cycloundecadiene, 8,8-dimethyl-9-methylene- | Hydrocarbons | 62338-54-9 | 0.38±0.02 | 0.26±0.02** | 1.77 | 0.69 |
| (1S,2E,6E,10R)-3,7,11,11-Tetramethylbicyclo[8.1.0]undeca-2,6-diene | Terpenoids | 24703-35-3 | 0.27±0.02 | 0.18±0.01** | 1.85 | 0.67 |
| 5-Hepten-2-ol, 6-methyl- | Alcohol | 1569-60-4 | 0.04±0.00 | 0.03±0.00** | 1.77 | 0.66 |
| Pentyl octanoate | Ester | 638-25-5 | 0.03±0.00 | 0.02±0.00** | 1.67 | 0.58 |

Note: The asterisk (*) in the same row indicates significant differences.(* $p < 0.05$ ** $p < 0.01$)

**Table S3.** Binding energies between key volatile compounds and olfactory receptors.

| Ligands | Binding energy (kcal/mol) | | | | |
|---|---|---|---|---|---|
| | OR1A1 | OR1D2 | OR1G1 | OR2W1 | OR5M3 |
| 1-Hexanol | -4.8 | -4.1 | -4.5 | -4.3 | -4.8 |
| 2-Nonenal, (E)- | -5.7 | -5.1 | -4.9 | -4.8 | -5.8 |
| 2-Propenoic acid, butyl ester | -5.4 | -4.6 | -4.6 | -4.9 | -5.4 |
| Benzene, n-butyl- | -6.6 | -6.6 | -5.5 | -6.1 | -7.1 |
| 3,6-Nonadien-1-ol, (E,Z)- | -6.4 | -5.9 | -5 | -5.3 | -5.7 |
| 3-Hexen-1-ol, (Z)- | -4.7 | -4.3 | -4.6 | -4.6 | -4.9 |
