# Supplementary figures and images for: Effects of Exogenous Tannase and Papain on the Flavor Quality of Black Tea During Fermentation
Source: Foods. 2026 May 14;15(10):1729. doi: 10.3390/foods15101729 (PMC13205800; doi:10.3390/foods15101729)

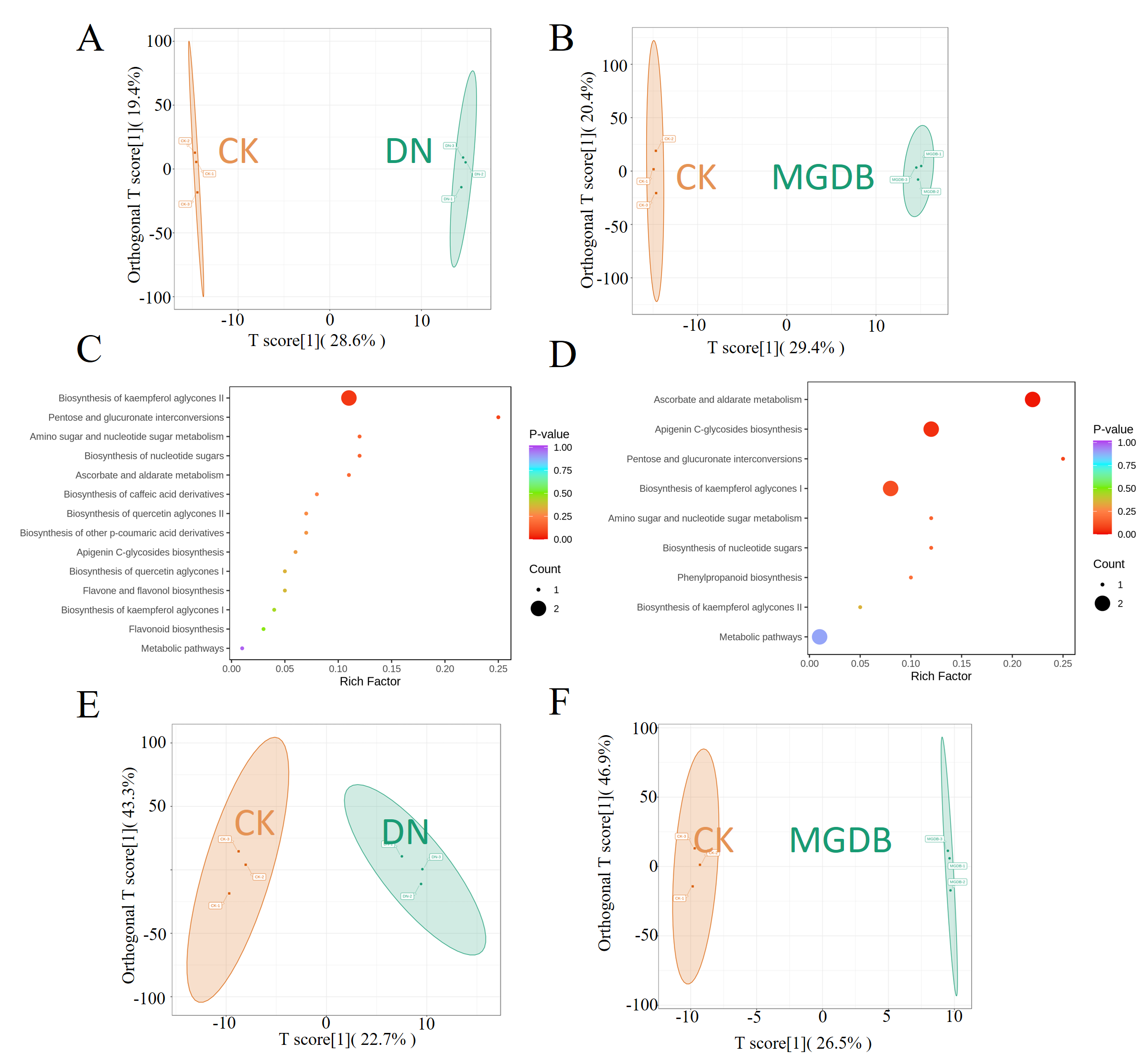

Supplement: Supplementary file 1 [file foods-15-01729-s001.zip › Figs. S1.bmp]
